# P-1126. Stratified Approach for Empiric Acyclovir in Neonates Presenting for Sepsis Evaluations

**DOI:** 10.1093/ofid/ofae631.1313

**Published:** 2025-01-29

**Authors:** Keerti L Dantuluri, Gang Liu, Nathaniel S O’Connell, Emily Que Tam Dellit, Pablo J Sanchez, Amina Ahmed

**Affiliations:** Levine Children's Hospital at Atrium Health, Charlotte, NC; Atrium Health, Charlotte, North Carolina; Wake Forest University School of Medicine, Winston Salem, North Carolina; Wake Forest University School of Medicine, Winston Salem, North Carolina; Nationwide Children's Hospital - The Ohio State University, Columbus, OH; Levine Children's Hospital at Atrium Health, Charlotte, NC

## Abstract

**Background:**

Neonatal herpes simplex virus (HSV) infection is rare but associated with high rates of morbidity. Recent guidelines recommend considering HSV in infants ≤ 21 days of age presenting for sepsis evaluations with certain risk factors. However, limited data exist on the value of these risk factors in stratifying the risk for neonatal HSV.

**Table 1:**
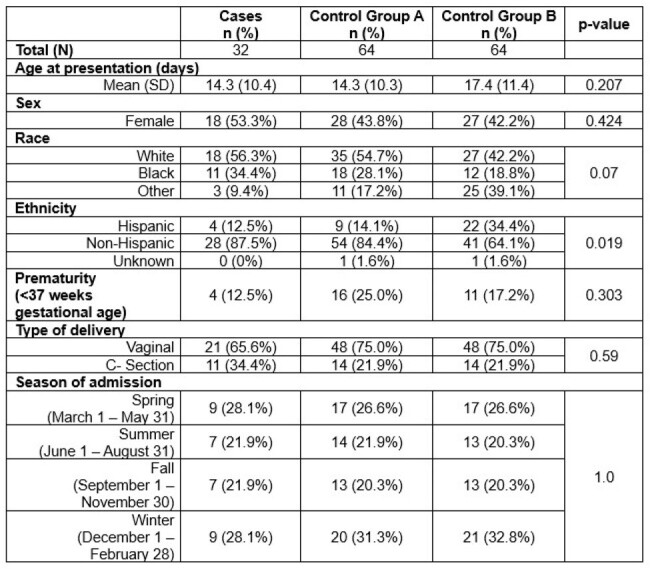
Characteristics of Cases and Controls

**Methods:**

We performed a retrospective case-control study of infants < 42 days of age who presented to Levine Children’s Hospital from 1/1/08 – 12/31/23 for sepsis evaluations to identify infants at low risk for neonatal HSV. We used Firth logistic regression to measure the odds ratio of the presence of high-risk criteria between infants without and those with HSV infection. High risk criteria included skin vesicles, seizures, elevated ALT, thrombocytopenia, CSF pleocytosis, and/or a sepsis-like picture. We matched each case with control patients without neonatal HSV admitted within 45 days of the case. For each case we identified 2 controls from group A, which included patients who tested negative for HSV, and 2 controls from group B, who may or may not have undergone HSV testing, but tested negative if they did (reflecting the general population of infants who present for sepsis evaluations).

**Table 2:**
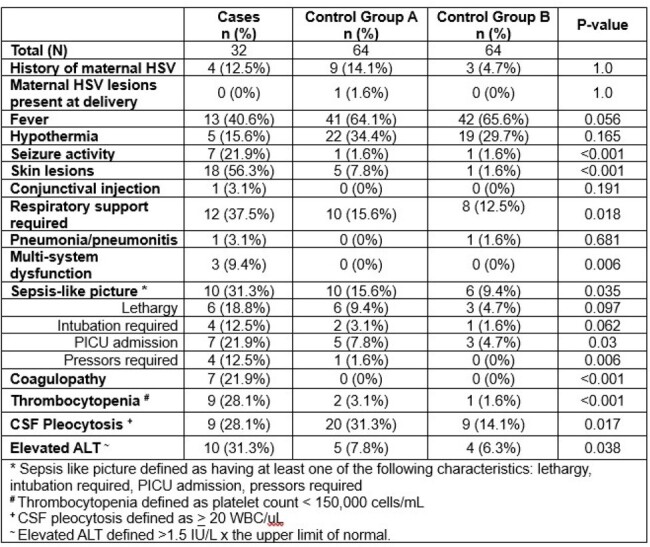
Clinical and Laboratory Presentation of Cases and Controls

**Results:**

Thirty-two cases and 128 controls (64 in group A and 64 in group B) were identified (**Table 1**). The mean age of cases was 14.3 days. The majority of cases were female, White, non-Hispanic, and born vaginally at term gestation. Controls, or infants without HSV, had lower prevalence of seizures, skin lesions, CSF pleocytosis, elevated ALT, thrombocytopenia, or sepsis-like picture compared to infants with HSV (**Tables 2 – 3**). The adjusted odds ratio of a control patient having at least one risk factor was 0.1 (95% CI 0.02 – 0.38) and 0.03 (95% CI 0 – 0.14) for control groups A and B, respectively, compared to cases (**Figure 1)**.

**Table 3:**
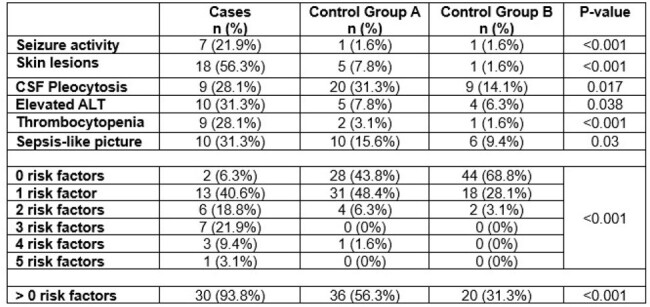
Prevalence of Risk Factors among Cases and Controls

**Conclusion:**

Among infants presenting for neonatal sepsis evaluations, those without HSV infection are less likely to present with seizures, skin lesions, elevated ALT, thrombocytopenia, CSF pleocytosis, or a sepsis like picture compared to infants with HSV. The lack of these criteria can be used to guide targeted evaluation and management of neonatal HSV to avoid unnecessary testing and treatment for low-risk infants.

**Figure 1:**
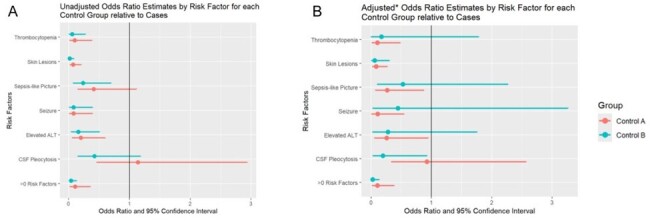
Odds Ratio Estimates by Risk Factor for Each Control Group Relative to Cases.

Orange bar represents control group A and blue bar represents control group B. Panel A depicts unadjusted odds ratio and panel B depicts adjusted odds ratios. Odd ratios in panel B are estimated from Firth logistic regression models and adjusted for age, race, sex, prematurity, type of delivery, season of admission, history of maternal HSV, and presence of maternal HSV lesions at delivery.

**Disclosures:**

**All Authors**: No reported disclosures

